# A bright cyan fluorescence calcium indicator for mitochondrial calcium with minimal interference from physiological pH fluctuations

**DOI:** 10.52601/bpr.2024.240001

**Published:** 2024-10-31

**Authors:** Wenjia Gu, Yuqin Yang, Yuqing Wang, Jia Li, Wanjie Li, Xiaoyan Zhang, Hao Dong, Youjun Wang

**Affiliations:** 1 Beijing Key Laboratory of Gene Resource and Molecular Development, College of Life Sciences, Beijing Normal University, Beijing 100875, China; 2 Kuang Yaming Honors School, Nanjing University, Nanjing 210023, China; 3 Joint Laboratory of Opto-Functional Theranostics in Medicine and Chemistry, The First Hospital of Jilin University, Changchun 130021, China; 4 State Key Laboratory of Analytical Chemistry for Life Science, Chemistry and Biomedicine Innovation Center (ChemBIC), & Institute for Brain Sciences, Nanjing University, Nanjing 210023, China; 5 Key Laboratory of Cell Proliferation and Regulation Biology, Ministry of Education, College of Life Sciences, Beijing Normal University, Beijing 100875, China

**Keywords:** Genetically encoded calcium indicator, mTurquoise2, pH, Mitochondria, MD simulations, *Ab initio* calculations

## Abstract

Genetically Encoded Calcium (Ca^2+^) indicators (GECIs) are indispensable tools for dissecting intracellular Ca^2+^ signaling and monitoring cellular activities. Mitochondrion acts as a Ca^2+^ sink and a central player for maintaining Ca^2+^ homeostasis. Accurately monitoring Ca^2+^ transients within the mitochondrial matrix that undergo constant pH fluctuations is challenging, as signals of most currently available GECIs suffer from artifacts induced by physiological pH variations. Multiplexed monitoring of optophysiology is also hindered by the limited availability of GECIs with cyan fluorescence. Based on the bright variant of cyan fluorescence protein (CFP), mTurquoise2, we developed a GECI designated as TurCaMP. Results from molecular dynamics simulations and *ab initio* calculations revealed that the deprotonation of the chromophore may be responsible for the Ca^2+^-dependent changes in TurCaMP signals. TurCaMP sensors showed inverse response to Ca^2+^ transients, and their responses were not affected by pH changes within the range of pH 6–9. The high basal fluorescence and insensitivity to physiological pH fluctuations enabled TurCaMP to faithfully monitor mitochondrial Ca^2+^ responses with a high signal-to-noise ratio. TurCaMP sensors allow simultaneous multi-colored imaging of intracellular Ca^2+^ signals, expanding the possibility of multiplexed monitoring of Ca^2+^-dependent physiological events.

## INTRODUCTION

Calcium ion (Ca^2+^) is an indispensable second messenger in eukaryotic cells (Berridge *et al.* 2003). Ca^2+^ signals regulate diverse processes such as synaptic transmission, gene transcription, fertilization, muscle contraction, and immune responses (Bollimuntha *et al.* 2017; Pan *et al.* 2014; Pinto *et al.* 2015; Shaw *et al.* 2012). Abnormal Ca^2+^ signaling is associated with disorders such as cancer, immune deficiency, and neurodegenerative diseases (Berna-Erro *et al.* 2012; Xie *et al.* 2015). As a central player in energy metabolism, the mitochondrion is also crucial in maintaining Ca^2+^ homeostasis (Malli and Graier 2017). However, accurate dissection of mitochondrial Ca^2+^ signaling is often hindered by pH fluctuations accompanying Ca^2+^ changes in the mitochondria matrix (Abad *et al.* 2004; Poburko *et al.* 2011).

Fluorescent Ca^2+^ indicators, especially Genetically Encoded Calcium indicators (GECIs), are essential tools for dissecting intracellular Ca^2+^ signaling (Roopa *et al.* 2019). GECI consists of a Ca^2+^-sensing moiety (sensor) and a fluorescence reporting module (reporter) (Gu *et al.* 2023). Ca^2+^ binding with its sensor domain would trigger fluorescent changes in the reporter (Kostyuk *et al.* 2019). For example, the most widely used single-fluorescent protein (FP)-type GECI, GCaMP series combines sensors such as calmodulin (CaM) and their target peptides (RS20 and others) with a reporter, circularly permuted enhanced green fluorescent protein (cpEGFP) (Akerboom *et al.* 2012; Baird *et al.* 1999; Chen *et al.* 2013; Dana *et al.* 2019; Tian *et al.* 2009; Zhao *et al*. 2011). To date, besides green-colored ones, GECIs with different fluorescence have been developed, such as red fluorescent RGECO1.2, jRGECO1a, yellow fluorescent XCaMP-Y, Inverse-Pericam and Y-GECO series, blue fluorescent XCaMP-B (Nasu *et al.* 2021). However, the pKa of the majority of these GECIs is close to the physiological pH (pH 7.4) (Suzuki *et al.* 2016). Thus, besides Ca^2+^, physiological pH variations may also modulate signals of these GECIs, resulting in undesired artifacts. It is necessary to develop novel GECIs that are less sensitive to fluctuation of cytosolic pH around pH 7.

To reveal the mechanism of Ca^2+^ signaling among organelles, and to dissect multiple neuronal ensembles underlying dynamic brain circuits, it is essential to develop multi-colored GECIs that enable multiplexed recording of either different subcellular compartments within single cells or multiple cell types in awake animals. However, not all colored GECIs are suitable or available for such purposes. For example, BFP-derived GECIs (Inoue *et al.* 2019; Zhao *et al.* 2011) are excited by ultraviolet light, which is highly phototoxic for live cells and has poor tissue-penetrating ability. These hurdles make them unsuitable for *in vivo* imaging (Icha *et al.* 2017). And the availability of the less phototoxic cyan-colored GECI is quite limited. A significant hurdle in developing GECIs with cyan fluorescence is that little is known about the molecular mechanisms underlying its Ca^2+^-dependent fluorescence changes and the lack of structural information (Akerboom *et al.* 2009). So far, only one mTurquoise2-derived GECI, Tq-Ca-FLITS (van der Linden *et al.* 2021) was reported. The basal fluorescence of Tq-Ca-FLITS is not very bright, and it is designed for equipment-demanding fluorescence lifetime imaging. Therefore, to fulfill the need for simultaneous multicolored imaging, like CFP/YFP/RFP, better performing cyan GECI with higher basal fluorescence are needed.

In the present study, we aimed to develop a novel cyan-colored GECI and provide some insight into the molecular mechanisms underlying its Ca^2+^ responses. Based on results from combined molecular dynamics (MD) simulation analysis together with experimental validation, we propose that changes in the extent of deprotonation of chromophore may be responsible for the modulation of cyan-colored GECI signals. We developed inverse cyan fluorescent GECIs, designated as TurCaMP, that are insensitive to physiological pH fluctuations. Compared to Tq-Ca-FLITS, TurCaMP indicators showed brighter basal fluorescence and a slightly larger dynamic range. We also proved that TurCaMP enables the monitoring of mitochondrial Ca^2+^ responses more accurately with a high signal-to-noise ratio. Even though there is still room for further optimization, TurCaMP sensors expand the possibility of multiplexed monitoring of Ca^2+^ signals and, hopefully, neural activities.

## RESULTS AND DISCUSSION

### Characterization of mTurquoise2-based GECI with GCaMP-like design (TurCaMP0.1)

**Figure 1:**
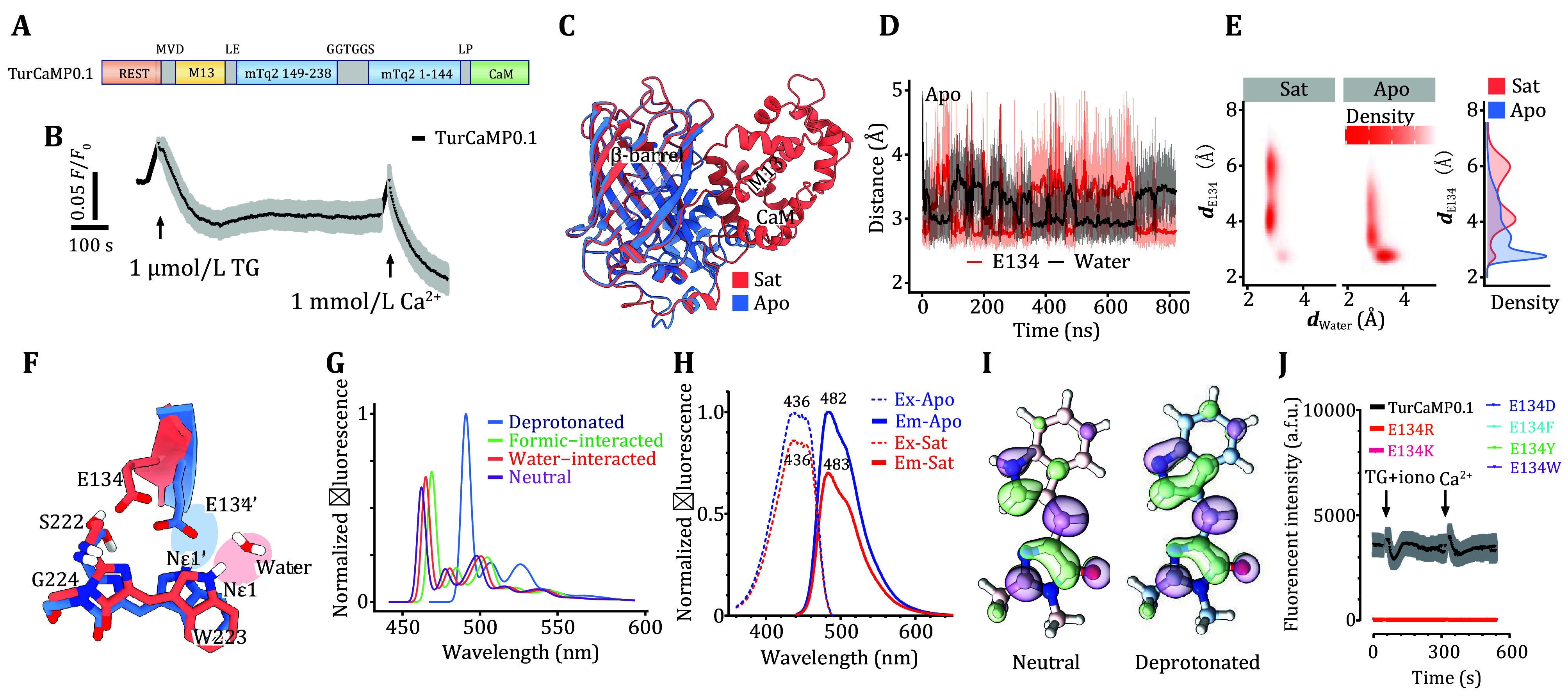
mTurquoise2-based GECI with GCaMP-like design (TurCaMP0.1) and its variants showed minimal Ca^2+^-dependent changes. **A** Diagram showing the design of the TurCaMP0.1 sensor, generated by replacing cpEGFP in GCaMP6m with circularly permutated mTurquoise2 (cpmTq2). **B** In HEK293 cells transiently expressing TurCaMP0.1, typical traces of Ca^2+^ release and subsequent SOCE responses induced by 1 μmol/L thapsigargin (TG) are shown (*n* = 3). **C–F** Molecular mechanism of Ca^2+^-triggered fluorescence modulation in TurCaMP0.1 as revealed by molecular dynamics (MD) simulations. Superposition of Ca^2+^-free TurCaMP0.1 (blue) and Ca^2+^-bound (red) aligned using the β-barrel segment. Two of the β-sheets are set to be transparent to avoid obscuring the chromophore (**C**). The N-O distance between Nε1 on the indole ring of the chromophore and the O atom of E134 or water (**D**). The probability density distribution of the N-O distances between the chromophore’s Nε1 and O of water & E134. Without Ca^2+^ binding (middle panel), a dominant distribution is attributed to E134 (highlighted in the bottom-right corner). The probability density distribution of the N-O distance between the chromophore Nε1 and the carboxylate moiety of E134 is shown in the left panel (**E**). Close-up view of the conformational differences around the chromophore in Panel C, indicating the assignment of water molecules to the Ca^2+^-bound structure. Structural elements and residue in the Ca^2+^-free TurCaMP0.1 structure are labelled with a prime symbol and color in blue. With Ca^2+^ bound, the chromophore interacts with water and color in red. In Ca^2+^-free conditions, the chromophore is more likely to interact with E134 (**F**). **G** Computed fluorescence spectra of the chromophore in different chemical environments and protonation states using *ab initio* calculations. **H** Fluorescence excitation and emission spectra of TurCaMP0.1 at pH 7.2, in the presence (red line, 39 μmol/L CaCl_2_) and absence (blue line, 10 mmol/L EGTA) of Ca^2+^. **I** The LUMO of the excited state of the chromophore. Both the neutral (left) and deprotonated (right) states of the chromophore are shown. **J** Typical traces showing TurCaMP0.1-E134 variants lost fluorescence and responsiveness to Ca^2+^ in HEK293 cells. Data were from three independent biological replicates, and traces are shown as mean ± SEM

To make a GECI with cyan (C) fluorescence protein (FP), we used a bright CFP variant, mTurquoise2 (Goedhart *et al.* 2012) (mTq2), as the template, replaced the circularly permutated (cp) enhanced green FP (cpEGFP) in GCaMP6m (Chen *et al.* 2013) with cpmTq2, and obtained the first version (0.1) of mTurquoise2-based GECI with GCaMP-like design, named TurCaMP0.1 (Fig. 1A and supplementary Table S1). When treated with a Ca^2+^ ionophore, ionomycin, to induce ER Ca^2+^ release, TurCaMP0.1 expressing cells showed a transient decline of mTq2 fluorescence. TurCaMP0.1 fluorescence also decreased in response to store operated Ca^2+^ entry (SOCE) following the addition of Ca^2+^ to extracellular bath solution (Fig. 1B). Thus TurCaMP0.1 could successfully report Ca^2+^ signals in live cells, even though the response amplitude was small.

To obtain TurCaMP variants with better performance, we set out to uncover the molecular mechanism of TurCaMP0.1. We conducted MD simulations on TurCaMP0.1 in both Ca^2+^-bound and Ca^2+^-free (apo) states, accumulating trajectories of over 3.5 μs for each system. The structural differences between TurCaMP0.1 in the two states are minimal (Fig. 1C), with a backbone root mean square deviation (RMSD) of ~1.8 Å. Additionally, in our simulations, the structural fluctuations of the β-barrel are quite small (supplementary Fig. S1), suggesting that the chemical environment surrounding the chromophore enclosed by the β-barrel only undergoes subtle changes.

Upon analyzing interactions around the chromophore, we observed that in the Ca^2+^-free state, a hydrogen bonding interaction exists between the Nε1 of the chromophore's indole group and the sidechain of E134. Simultaneously, water molecules in the vicinity compete with E134 for interaction with the chromophore's Nε1, and this competition can switch rapidly on the order of tens of nanoseconds (Fig. 1D). The intrinsic protein dynamics have also been observed in theoretical studies of other fluorescent proteins (Deng *et al.* 2021; Lelimousin *et al.* 2009). As shown in Figs. 1E and 1F, following the binding of Ca^2+^ ions, interactions between water molecules and the chromophore dominate; in contrast, hydrogen bonds between E134 and the chromophore are rarely observed. Therefore, we propose that in TurCaMP0.1, there are two distinct interaction modes for the chromophore. In the presence of Ca^2+^ ions, the chromophore stably interacts with surrounding water molecules (Fig. 1F, red). Conversely, in the absence of Ca^2+^ ions, in addition to interactions with water, the chromophore forms hydrogen bonds with E134 (Fig. 1F, blue), which can activate the proton in indole. Furthermore, E134 interacts with S222 through hydrogen bonding in both systems, as reported in mTurquoise (Goedhart *et al.* 2012), stabilizing the chromophore interaction.

To gain further insights into the regulatory mechanisms of these two interaction modes on fluorescence properties, we constructed a chromophore model and utilized *ab inito* calculations to compute the fluorescence spectra of the chromophore under different interaction modes. As shown in Fig. 1G, the calculated fluorescence spectrum of the isolated chromophore exhibited a maximum fluorescence emission wavelength of 462 nm, with a difference of 20 nm from the experimentally determined value (482 nm) (Fig. 1H). Furthermore, we conducted calculations on the fluorescence spectra of the chromophore when it forms hydrogen bonds with either water molecules or formic acid, which is used to simulate the effect of the carboxylate group of E134. The results indicated that in the presence of water and carboxylate group, the fluorescence intensity increased by 9% and 14%, respectively, while the maximum fluorescence emission wavelengths red-shifted to 465 nm and 469 nm, respectively. When the chromophore interacted with the water molecule or formic acid, the hydrogen atom Hε1 became partially activated, leading to an increase in the Nε1-Hε1 distance from 1.009 Å to 1.023 and 1.040 Å, respectively. Given that classical MD simulations cannot capture proton transfer processes, we employed *ab initio* calculations to determine the energy barrier for proton transfer from the chromophore to E134, which was found to be 3.2 kcal/mol (supplementary Fig. S2). This suggests that in the presence of the carboxylate group, the chromophore may shift toward a deprotonated state. We then computed the fluorescence spectrum of the deprotonated chromophore and found a 64% enhancement in fluorescence compared to the neutral chromophore. This dynamic range agrees with experimental observations (Fig. 1B). As shown in Fig. 1I, the excited-state lowest unoccupied molecular orbital (LUMO) of the deprotonated chromophore exhibits higher electron delocalization than the neutral form. These differences in electronic structure led to changes in fluorescence intensity.

Based on modeling and experimental observations, we propose the regulation mechanism of TurCaMP0.1. In the absence of Ca^2+^, the two interaction modes of chromophore coexist: interaction with E134 through hydrogen bonding, and interaction with water. E134 interacts with the chromophore through hydrogen bonding, activating the chromophore's Hε1 and making it more likely to be fully deprotonated, resulting in strong fluorescence, with a peak around 491 nm (Fig. 1G). The enhancement of fluorescence by the deprotonation state of the chromophore has also been reported in the GFP system (Striker *et al.* 1999). On the other hand, another concurrent interaction involves water molecules interacting with the chromophore. In this case, the proton activation of the chromophore is lower, resulting in lower calculated fluorescence intensity, and the fluorescence wavelength is around 465 nm. The coexistence of these two modes has been experimentally validated. It has been observed that TurCaMP0.1, in the absence of Ca^2+^, exhibits two peaks around 483 nm and 510 nm. Upon binding of calcium ions, the movement of the M13-like domain relative to the CaM calcium-sensing domain affects the water environment around the chromophore. This allows water to interact more strongly with the chromophore, competitively disrupting the interaction between the chromophore and E134. As a result, the interaction between the chromophore and E134, which results in stronger fluorescence, shows a significant reduction in proportion (Fig. 1E). This explains the experimentally observed weaker fluorescence in the calcium-bound state. The water-mediated regulation mechanism of the chromophore has also been reported in NCaMP7 (Subach *et al.* 2020). We further confirmed the role of E134 in fluorescence enhancement through experiments by checking the effects of E134 mutation on Ca^2+^ responses. Substitution of E134 with residues bearing different charges, polarity, or sizes of side chains resulted in a complete loss of fluorescence in TurCaMP (Fig. 1J). Thus, the E134 is conserved, and no further attempts were made to optimize TurCaMP0.1.

### Screening and performance of TurCaMP variants with NEMO-like design

**Figure 2:**
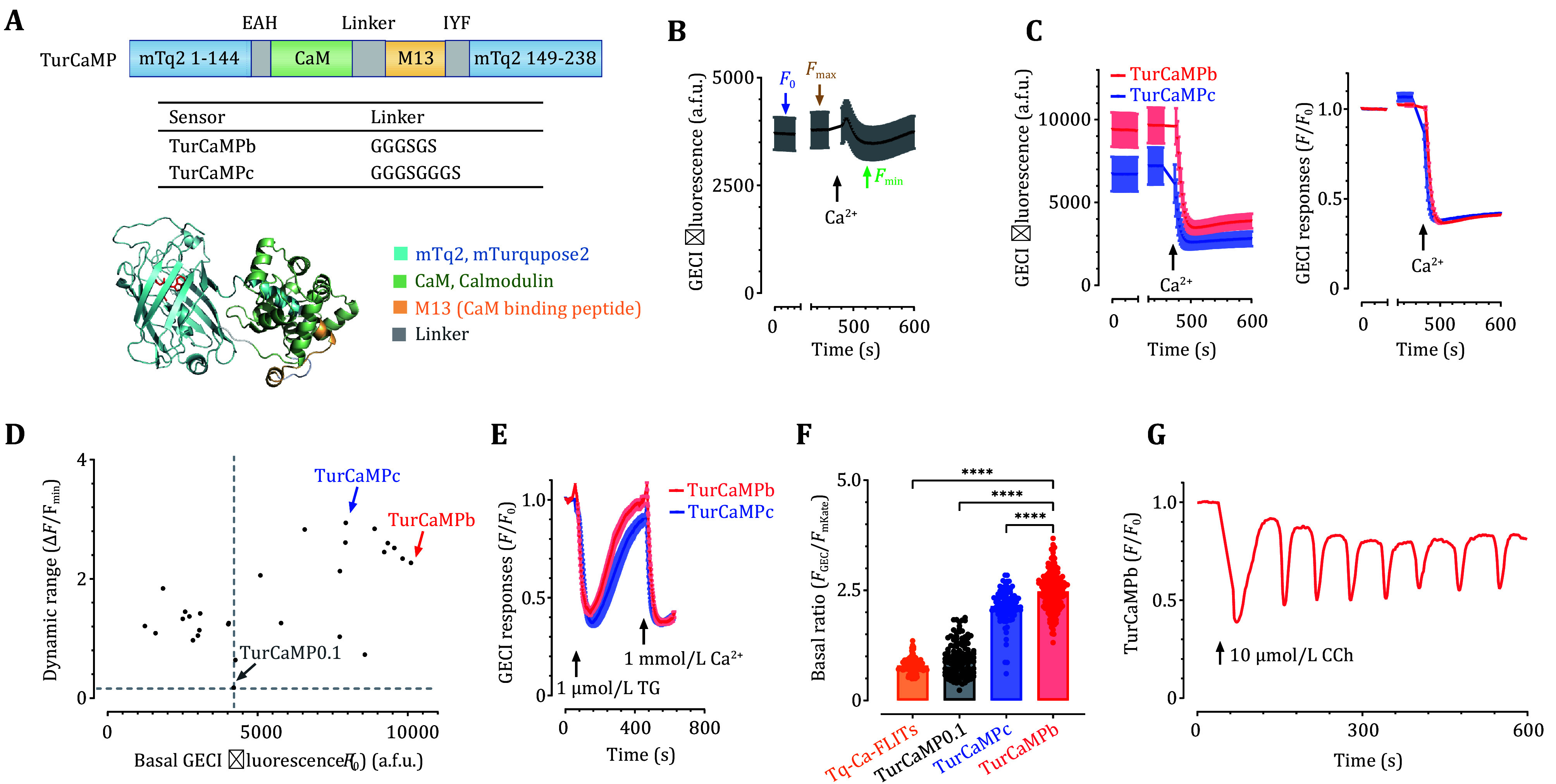
*In cellulo* screening and performance of optimized TurCaMP indicators with a NEMO-like design. **A** TurCaMP sensors engineered with the NEMO-like design. Coding sequences of mNeonGreen fragments within NEMOm were replaced with those of the corresponding mTq2 domain. Top and middle panels, a diagram showing the design of TurCaMP variants; the bottom panel, the structure of TurCaMP predicted with I-TASSER. **B**–**D** Screening of TurCaMP variants with Ca^2+^ imaging using HEK293 cells. A typical trace showing the screening assay based on Ca^2+^ imaging. After the recording of basal fluorescence (*F*_0_) of cells transiently expressing TurCaMP0.1, the endoplasmic reticulum Ca^2+^ store was depleted using 2.5 μmol/L ionomycin (iono) and 1 μmol/L TG imaging solution to obtain maximal GECI fluorescence (*F*_max_). Finally, Store-operated Ca^2+^ entry was induced by the addition of 30 mmol/L Ca^2+^ to obtain the minimal response (*F*_min_) (**B**). Typical fluorescent intensity traces (left panel) and relative fluorescence traces (right panel) of TurCaMPb and TurCaMPc (**C**). Scatter plot of *F*_0_–mean dynamic range ((*F*_max_ – *F*_min_)/*F*_min_) of TurCaMP variants (**D**). **E** Ca^2+^ release and store-operated Ca^2+^ entry (SOCE) responses induced by 1 μmol/L TG in HEK293 cells were compared between TurCaMPb and TurCaMPc. **F** Basal brightness of TurCaMP sensors. To exclude artifacts caused by differences in expression, the basal fluorescence of GECIs (*F*_GECI_) of cells expressing mKate-P2A-GECI constructs was normalized against the fluorescence of mKate, an expression marker (*F*_mKate_). (****P <0.0001, unpaired Student’s *t*-test, two-tailed). **G** Typical trace of Ca^2+^ oscillations induced by 10 μmol/L carbachol (CCh) detected by TurCaMPb in HEK293 cells. Data were from three independent biological replicates, and error bars denote SEM

Since TurCaMP0.1 indicators showed small dynamics and simulation-guided optimization failed to work, we switched to a NEMO-like design by inserting the CaM-M13 Ca^2+^ sensing module into mTq2 (Fig. 2A). Using NEMO sensors (Li *et al.* 2023) as templates, we generated a small pool of TurCaMP variants by replacing mNeonGreen fragments in NEMO with those of mTq2 (Fig. 2A and supplementary Table S2), transfected them into HEK293 cells, and subsequently screened their fluorescence at basal (*F*_0_), maximal (*F*_max_) and minimal (*F*_min_) conditions with a previously established *in cellulo* Ca^2+^ imaging assay (Li *et al.* 2023) (Fig. 2B). Briefly, after obtaining *F*_0_, cellular Ca^2+^ was depleted by 10-min incubation with 2.5 μmol/L ionomycin and 1 μmol/L thapsigargin (TG, an inhibitor of the sarcoplasmic/endoplasmic reticulum Ca^2+^ ATPase) to get *F*_max_. In the end, a high amount of Ca^2+^ (30 mmol/L) was added to the bath solution to reach *F*_min_ via SOCE. Corresponding changes of raw (Fig. 2C, left panel) or relative changes in fluorescence (Fig. 2C, right panel) were then used to calculate the dynamic range (*DR* = (*F*_max_ – *F*_min_) / *F*_min_). Based on the *F*_0_ and large DR values of screened TurCaMP constructs (Fig. 2D), the high contrast TurCaMPc and bright TurCaMPb for further testing.

We next examined the performance of TurCaMP in detecting TG-induced Ca^2+^ releases and SOCE signals, two types of relatively small Ca^2+^ responses (Prakriya and Lewis 2015) in HEK 293 cells (Fig. 2E). The results showed that TurCaMP signals decreased more than 60% upon addition of TG. Since TG-induced Ca^2+^ releases and SOCE responses shown by TurCaMPc and TurCaMPb are similar in amplitudes (*P* = 0.292 and 0.334, respectively), and TurCaMPb had brighter basal fluorescence (Fig. 2D), we focused on TurCaMPb for further investigation. Indeed, the basal brightness of TurCaMPb is approximately 3.1-folds of Tq-Ca-FLITs and 2.6-folds of TurCaMP0.1 (Fig. 2F). This bright basal signal makes it suitable for visualizing Ca^2+^ transients within small subcellular compartments like dendritic spines or mitochondria. The responses of TurCaMP to submaximal activation of muscarinic acetylcholinergic receptors with carbachol (CCh, 10 μmol/L) were examined in HEK293 cells. It is well established that 10 μmol/L CCh could induce cytosolic Ca^2+^ oscillations (Dupont *et al.* 2011), and TurCaMPb showed a robust oscillatory signal (Fig. 2G). Together, these results established that the TurCaMP indicator is a cyan-colored GECI capable of reporting cytosolic Ca^2+^ signals induced by various stimuli.

### *In vitro* characterization of TurCaMPb

**Figure 3:**
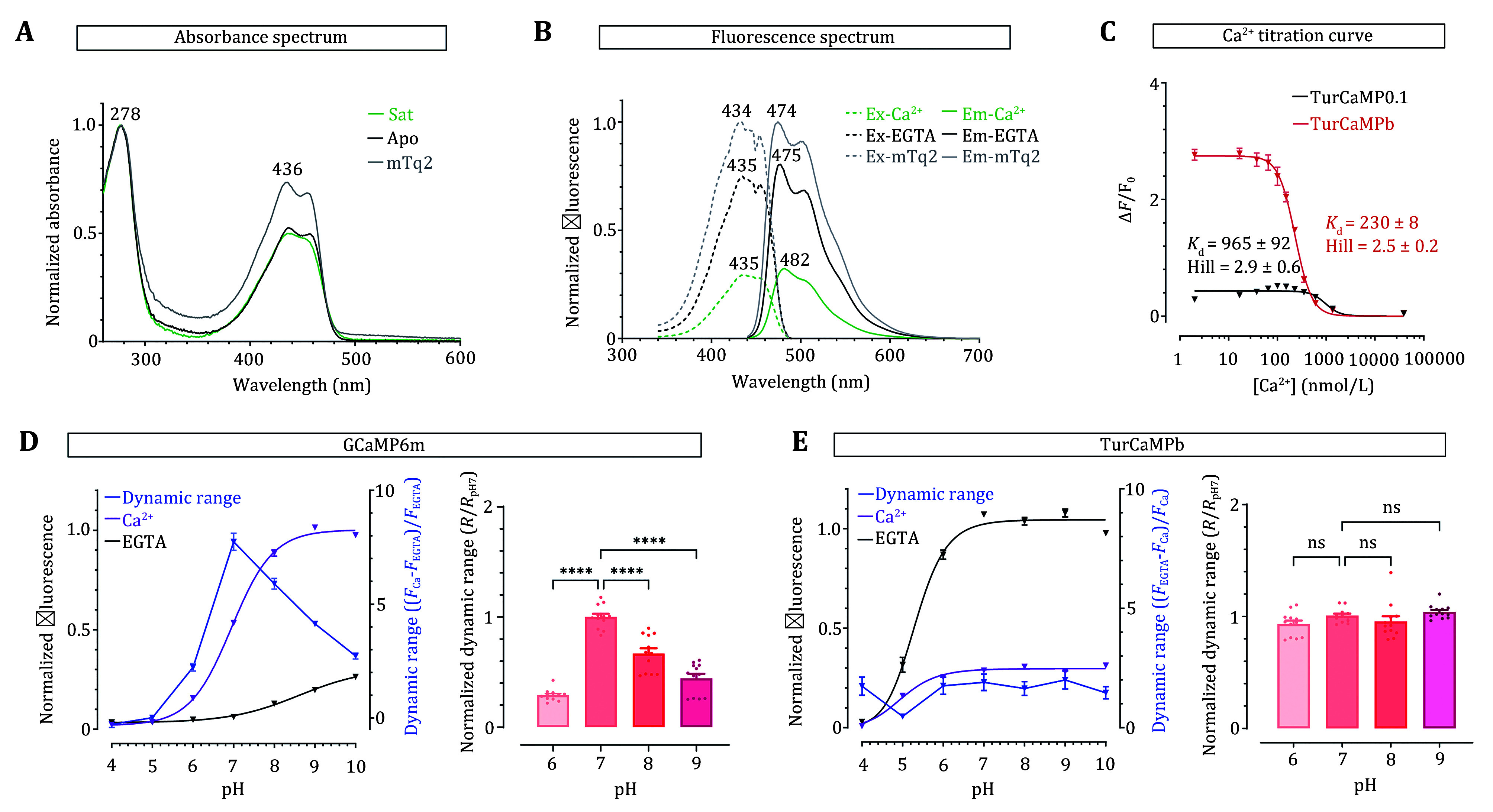
*In vitro* characterization of TurCaMPb. **A** Absorbance spectrum of TurCaMPb in the presence (green line, 39 μmol/L CaCl_2_) and absence (black line, 10 mmol/L EGTA) of Ca^2+^ compared with mTq2 (gray line). **B** Fluorescence excitation (dashed line) and emission (solid line) spectra of TurCaMPb, with presence (green line) and absence (black line) of Ca^2+^ compared with mTq2 (gray line). **C** Ca^2+^ titration curves of TurCaMP0.1 and TurCaMPb. **D**,**E** pH titration responses of GCaMP6m (**D**) or TurCaMPb (**E**). Left, typical traces, with purple line showing the responses of Ca^2+^-bound GECI (*F*_Ca_), the black traces representing behaviors of GECIs in Ca^2+^-free traces (*F*_EGTA_), and the blue curves showing the dynamic range. Right, statistics changes of *F*_GECI_ at different pH relative to that at pH 7 (Right column in Panel D, ****P < 0.0001; Right column in Panel E, pH 7 vs. 6, *P* = 0.2228, pH 7 vs. 8, *P* = 0.5037, pH 7 vs. 9, *P* = 0.7927; One-way ANOVA). Data were from three independent replicates, and error bars denote SEM

The spectral properties of Ca^2+^-bound TurCaMPb stay similar to its template mTq2, both showed dual fluorescence emission peaks (Figs. 3A and 3B), suggesting that they may correspond to different proton activation degrees of the chromophore, as indicated by the aforementioned computational results obtained with TurCaMP0.1 (Fig. 1G). Unlike TurCaMP0.1, the peak emission of TurCaMPb is much bigger at 510 nm (Fig. 3B), probably an indication of stronger interaction with E222 residue (corresponding to E134 residue in TurCaMP0.1), resulting in brighter basal fluorescence (Fig. 2F). This 510 nm peak largely diminished in Ca^2+^-free state (Fig. 3B), possibly leading to a decrease in fluorescence caused by weaker interaction with E222 residue. Unfortunately, there is no structural information available for the apo state of NCaMP7-like design, preventing us from performing MD simulations to check whether TurCaMPb adapts a similar Ca^2+^-dependent modulation of its fluorescence. Further investigations are needed to elucidate this phenomenon.

We next assessed the molecular fluorescence brightness of TurCaMPb by determining the quantum yields (QY) and extinction coefficient in the saturated (sat) and apo states (supplementary Table S3). The measured change in its brightness was consistent with in-cell data obtained from HEK293 cells (*F*_apo_ / *F*_sat_ = 3.27). The results also demonstrated that, upon Ca^2+^ binding, its extinction coefficient remained relatively constant, while the QY of TurCaMPb exhibited a 54% decline. Since the lifetime of fluorescence protein is proportional to its QY, the relatively high Ca^2+^-dependent QY contrast of TurCaMPb may enable quantitative detection of Ca^2+^ signals using fluorescence lifetime imaging microscopy (FLIM) (van der Linden *et al.* 2021).

We further determined the Ca^2+^ affinities of TurCaMP sensors *in vitro*. Unlike TurCaMP0.1 that has a dissociation constant in the sub-micro molarity range (0.97 ± 0.09 μmol/L), TurCaMPb showed a *K*_d_ of approximately 0.23 μmol/L, making it suitable for detecting Ca^2+^ fluctuations around basal cytosolic Ca^2+^ levels (Fig. 3C and supplementary Table 3).

It was reported that mTq2 has a p*K*a of 3.1, making it very resistant to acidic conditions (Emrich *et al.* 2021; Goedhart *et al.* 2012; Wedel *et al.* 2007). Using GCaMP6m as a control, we thus checked whether TurCaMPb and TurCaMP0.1 retain this acid-insensitive property by examining its pH-dependent fluorescence changes in its apo or Ca^2+^-bound state. Similar to previous reports, the p*K*a of GCaMP6m is around 7.8 (Chen *et al.* 2013), close to resting cytosolic pH (7.4). Thus, when pH varies within 6–9, the fluorescence and dynamic range of GCaMP6m change significantly (Fig. 3D). The property makes GCaMP indicators less desirable for detecting Ca^2+^ signals under pH-varying circumstances (Pendin *et al.* 2015). In sharp contrast, TurCaMPb and TurCaMP0.1 fluorescence, both at its apo and Ca^2+^-bound state, stays stable in the pH range of 7–9 (Fig. 3E and supplementary Fig. S3). As a result, the dynamic range of TurCaMPb did not significantly change with pH under physiological conditions (Fig. 3E), making it suitable for monitoring Ca^2+^ signals in a pH-varying environment like in the mitochondrial matrix.

### Monitoring of mitochondrial Ca^2+^ signals with TurCaMPb

**Figure 4:**
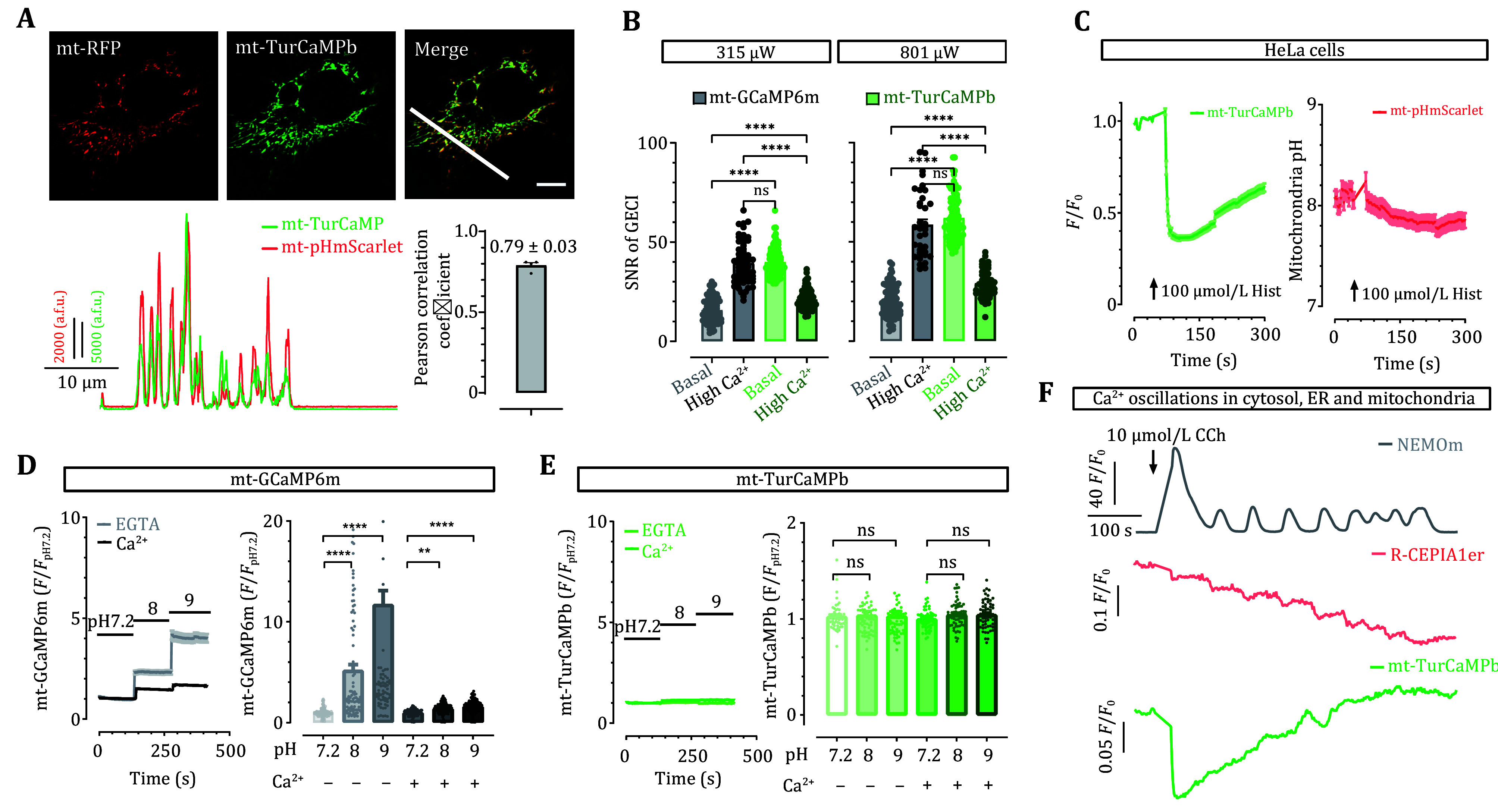
Subcellular imaging of Ca^2+^ signals with TurCaMPb and other GECIs. **A** mt-TurCaMPb co-localized well with mt-RFP, a mitochondrial marker, in HEK293 cells. Top panel, a typical confocal image showing the co-localization between mt-TurCaMPb (green) and mt-RFP (red). Scale bar: 10 μm. Bottom left panel, intensity plots representing the co-localization of mt-TurCaMPb and mt-RFP. Bottom right panel, statistics. **B** In HeLa cells transiently expressing mt-TurCaMPb or mt-GCaMP6m, upon illumination with 315 μW or 801 μW light, the mt-TurCaMPb signal showed higher basal SNR than that of mt-GCaMP6m. (****P < 0.0001; ns, not significant, for 315 μW, ns, *P* = 0.8742, for 801 μW, ns, *P* = 0.2300; Two-way ANOVA). **C** Mitochondrial acidification accompanied by 100 μmol/L histamine-induced elevations of [Ca^2+^]_mt_ in HeLa cells. In cells transiently co-expressing mt-TurCaMPb and mt-pHmScarlet, a pH indicator for mitochondrial matrix, changes in [Ca^2+^]_mt_ and [pH]_mt_ were indicated by mt-TurCaMPb and mt-pHmScarlet, respectively. **D**,**E** Unlike mt-GCaMP6m, *in cellulo* mt-TurCaMPb signal was insensitive to tested pH fluctuation in HeLa cells. Typical pH responses of mt-GCaMP6m (**D**) and mt-TurCaMP (**E**), correspondingly. Right, statistics. **P = 0.0016, ****P < 0.0001 (**D**), pH 7.2 vs. 8 without Ca^2+^, *P* = 0.9458, pH 7.2 vs. 9 without Ca^2+^, *P* = 0.9963, pH 7.2 vs. 8 with Ca^2+^, *P* = 0.1102, pH 7.2 vs. 9 with Ca^2+^, *P* = 0.1041; One-way ANOVA (**E**). **F** Typical multiplexed recording of CCh (10 μmol/L) induced Ca^2+^ responses within the cytosol (by NEMOm, gray line), ER (by R-CEPIAR1er, red line) and mitochondria (by mt-TurCaMPb, green line). Data were from three independent biological replicates, and curves are shown as mean ± SEM

By adding the tandem repeat of the mitochondrial matrix targeting sequence, cytochrome C oxidase subunit VIII (COX VIII), to the N-terminus of TurCaMPb, we constructed mitochondrial TurCaMPb (mt-TurCaMPb). When co-expressed with a fluorescent mitochondrial maker, mt-RFP, mt-TurCaMPb showed good colocalization with mt-RFP (Fig. 4A). We next checked whether mt-TurCaMPb could specifically detect histamine-induced Ca^2+^ increases within matrix of mitochondria by examining the effects of blocking mitochondrial-Ca^2+^-uptake with 10 μmol/L carbonyl cyanide 4-(trifluoromethoxy) phenylhydrazone (FCCP), an uncoupler of the mitochondrial respiratory chain (Ishii *et al.* 2006). In HeLa cells transiently expressing mt- TurCaMPb, stimulation with 100 μmol/L histamine could induce a robust decrease in mt-TurCaMPb fluorescence (supplementary Fig. S4). Consistent with reports showing FCCP will not inhibit cytosolic Ca^2+^ increases, only block mitochondrial Ca^2+^ uptake, 10 μmol/L FCCP diminished histamine-induced decreases of mt-TurCaMPb signal, demonstrating that mt-TurCaMPb indeed is a reliable GECI for mitochondria.

As aforementioned, mt-TurCaMPb is an inverse-responding GECI with high basal fluorescence, likely enabling the detection of Ca^2+^ signals within small subcellular compartments with high signal-to-noise ratio (SNR). We thus compared the basal SNR values of mt-TurCaMPb with mt-GCaMP6m, a mitochondria-targeting GECI. The results demonstrated that, under illuminations with the same power, mt-TurCaMP did show significantly higher basal SNR than that of mt-GCaMP6m (Fig. 4B). The SNR values of both GECI probes were also measured under high Ca^2+^ states, by incubating store-depleted cells in 30 mmol/L Ca^2+^ to maximize mitochondrial Ca^2+^ levels via SOCE. Since mt-TurCaMPb is an inverse GECI, the SNR of these two indicators were compared under fluorescence high or low conditions, respectively. The results showed that mt-TurCaMPb exhibited higher SNR under corresponding low fluorescence conditions. This property thus allows imaging of mitochondria with low intensity of excitation light, resulting in lower light toxicity as compared with other cyan-colored GECI (Icha *et al.* 2017).

It is well established that changes in Ca^2+^ levels within the mitochondrial matrix are often accompanied by variations in pH (Abad *et al.* 2004; Hou *et al.* 2017; Poburko *et al.* 2011). Thus, a mitochondria-localized pH sensor, mt-pHmScarlet (Liu *et al.* 2021), was made and confirmed to co-localize well with a BFP-colored mitochondrial marker, mt-BFP (Pearson correlation coefficient = 0.814 ± 0.031). We then co-expressed mt-TurCaMPb and mt-pHmScarlet in mammalian cells, and simultaneously monitored histamine-induced changes in both pH and Ca^2+^ levels. Similar to previous reports (Poburko *et al.* 2011), the results demonstrated that 100 μmol/L histamine decreased fluorescence of both mt-TurCaMPb and mt-pHmScarlet (Fig. 4C), showing that increases in mitochondrial Ca^2+^ levels are indeed accompanied with decreases in pH. Consistent with results from *in vitro* experiments (Fig. 3D), mt-GCaMP6m signal is significantly altered by changes in pH *in situ* (Fig. 4D), thus mitochondrial Ca^2+^ transients reported with GCaMP type GECI contains intrinsic artifacts caused by pH alterations. Contrary to GCaMP sensors, mt-TurCaMPb signals do not change with pH fluctuations within the range of 7.2–9 (Fig. 4E). Thus, this insensitivity to physiological pH variations makes mt-TurCaMPb a reliable reporter for mitochondria Ca^2+^ signals.

Lastly, with the help of this cyan-colored mt-TurCaMPb, we used CFP/YFP/RFP filter sets to simultaneously monitor Ca^2+^ changes in ER, cytosol and mitochondria during CCh-induced Ca^2+^ oscillations (Fig. 4F). In HEK R-CEPIA1er stable cells transiently co-expressing NEMOm and mt-TurCaMPb, 10 μmol/L CCh successfully induced cytosolic Ca^2+^ oscillations shown by NEMOm (gray trace). Consistent with previous reports, these oscillatory Ca^2+^ signals mostly arise from repetitive ER Ca^2+^ emptying and refilling, as demonstrated by R-CPEIA1er oscillations (red trace). Meanwhile, Ca^2+^ ions released into the cytosol are taken up into mitochondria, as reflected by decreases in mt-TurCaMPb signals (green trace).

Overall, we generated a cyan-colored, inverse GECI designated as TurCaMPb for accurately monitoring Ca^2+^ transients in small subcellular compartments showing frequent pH fluctuations in the range of pH 7–8. Even though the dynamic range of TurCaMPb still needs further optimization, it may help better dissect mitochondrial Ca^2+^ signaling.

### Experimental procedures

#### Plasmid construction

To construct TurCaMP0.1, we first amplified coding sequences of mTurquoise2 (mTq2) 1–144 and 149–238 from that of mTurquoise2 via PCR to generate circularly permutated mTq2 (cpmTq2), then we replaced cpEGFP in GCaMP6m with cpmTq2 and obtained TurCaMP0.1. To make other TurCaMP variants, we replaced the corresponding coding sequence of mNeonGreen fragments within NEMO indicators (Li *et al.* 2023) with those of mTq2_1–144_ and mTq2_149–238_. To construct mitochondria-targeted indicators, the tandem repeat of mitochondrial matrix targeting sequence, cytochrome C oxidase subunit VIII (COX VIII), was added to the N-terminus of pHmScarlet (kindly provided by Prof. Pingyong Xu), TurCaMP or GCaMP6m. To construct mKate-P2A-GECI indicators, we replaced NEMO in mKate-P2A-NEMO plasmids (Li *et al.* 2023) with TurCaMP variants and Tq-Ca-FLITs. All fragments were inserted into the pCDNA3.1(+) vector between BamHI and EcoRI sites by using a Ready-to-Use Seamless Cloning Kit (Sangon Biotech, Shanghai, China). To make plasmids for bacterial expression, the sequences of TurCaMP0.1 and TurCaMP variants were amplified with PCR and inserted into a pET28a vector linearized by NcoI and XhoI with the help of a Ready-to-Use Seamless Cloning Kit. mTq2 and Tq-Ca-FLITs were synthesized by BGI Geneland Scientific Co., Ltd, China. mt-RFP and mt-BFP were kindly provided by Prof. Junjie Zhang. All plasmids were confirmed by Sanger’s sequencing.

#### Bacterial expression and protein purification

TurCaMP0.1- or TurCaMP-encoding pET28a plasmids were transformed into Transetta (DE3) bacteria (Transgene, Beijing, China). Transformed bacteria were first grown in LB medium supplemented with 100 μg/mL kanamycin till the medium reached an OD of 0.6 (37 °C, 200 r/min). Then 300 μmol/L IPTG was added and cells were cultured at 20 °C for another 9 h (150 r/min) to induce protein expression. Afterwards, cells pellets collected with centrifugation were re-suspended and lysed with buffer 1 (in mmol/L, 20 Tris, 300 NaCl, 1 imidazole, pH = 7.2) containing 1 mg/mL lysozyme, 8 mmol/L PMSF and 0.5 % Triton X-100. The resulting preparation was sonicated, and centrifuged (4 °C, 9000 r/min, 20 min) to extract total protein from supernatant. Total protein was then applied to 1 mL Ni Sepharose column (17-5318-01, GE Healthcare, Piscataway, NJ, USA) to enrich GECIs with histidine tags. Next, columns were washed with 20 mL buffer 1 and 10 mL buffer 2 (in mmol/L, 20 Tris, 500 NaCl and 10 imidazole, pH 7.2). Purified GECI proteins were eluted with 5 mL buffer 3 (in mmol/L, 20 Tris, 100 NaCl and 300 imidazole, pH 7.2) (Li *et al.* 2023).

#### System Setup and MD simulations

For the Ca^2+^-bound structure, two structures were used as templates for homology modeling: one is the X-ray crystal structure of Ca^2+^-bound GCaMP2 (Akerboom *et al.* 2009) (PDB entry: 3EK4), and the other is the Ca^2+^-bound structure of GCaMP6m (Ding *et al.* 2014) (PDB entry: 3WLD). The X-ray crystal structure of GCaMP2 in the absence of Ca^2+^ (PDB entry: 3EKJ) (Akerboom *et al.* 2009) was used for homology modeling to construct the Ca^2+^-free TurCaMP0.1 structure. Then, the constructed protein was solvated into bulk water by adding water molecules around the protein in all three dimensions (x, y, and z) with a padding of 15 nm. A 150 mmol/L concentration of CaCl_2_ was added, with a few more ions to neutralize the system, to mimic the physiological condition of salt concentration. This system contains ~75,000 atoms, including ~23,000 water molecules.

For both systems, energy minimization was first used to remove bad contacts, where harmonic restraints were applied on the protein backbone atoms. The simulation system was then equilibrated, with gradually decreased harmonic position restraints applied to the heavy atoms of the protein backbone. After equilibration, the simulations were continued in the NPT ensemble at 1 atm pressure and 310 K for at least 800 ns. The first 100 ns of each trajectory was not used for analysis. We carried out at least three replicate MD simulations for each system and obtained consistent results. The RMSD for each trajectory is shown in supplementary Fig. S1. The structures obtained from homology modeling based on 3EK4 showed relatively substantial variations in RMSD. Therefore, only the trajectories simulated using the homology modeling structure derived from 3WLD were utilized as the Ca^2+^-bound structure for analysis. The MD simulations were carried out using the CUDA-accelerated NAMD program version 2.14 (Phillips *et al.* 2020). The CHARMM36 force field parameters (Best *et al.* 2012) and the TIP3P water model (Jorgensen *et al.* 1983) were used. Periodic boundary conditions were applied, and the particle mesh Ewald method (Darden *et al.* 1993) was used to treat long-range electrostatic interactions. VMD (Humphrey *et al.* 1996) was used to analyze MD trajectories. UCSF ChimeraX (Goddard *et al.* 2018) was used to visualize the models.

#### Ab Initio Calculations

*Ab initio* calculations were performed using Gaussian16 (Frisch *et al.* 2016). The initial structures were extracted from the trajectories of MD simulations. The ground state structures were optimized, and their frequencies were calculated at the B3LPY/6-311+G(d) (Becke 1993; Hehre *et al.* 1969) level. The solvent effect was considered with the polarized continuum model (PCM) (Tomasi *et al.* 2005). Subsequently, the excited-state structures were optimized based on the corresponding ground-state configurations, and the subsequent frequency calculations were performed at the same computational level using the time-dependent density functional theory (TDDFT) method. The vibrationally resolved fluorescence spectra were computed using the Franck-Condon (FC) principle.

#### Biophysical properties of purified proteins

To measure spectral properties of TurCaMPb and TurCaMP0.1, purified proteins were dissolved in solution (100 mmol/L KCl, 30 mmol/L MOPS, pH = 7.2, with either 10 mmol/L EGTA or 10 mmol/L Ca-EGTA). Absorption spectrums were measured by a spectrophotometer (Model UV2600, Shimadzu, Japan). Fluorescence spectrums were measured by a fluorescence spectrophotometer (FS5, Edinburgh Instrument, Scotland).

To assess the dissociation constant for Ca^2+^ (Tsien and Pozzan 1989), the purified proteins (50 μg/mL) were added into various buffered Ca-EGTA solutions (100 mmol/L KCl, 30 mmol/L MOPS, pH = 7.2) that contain 11 different free Ca^2+^ concentrations, ranging from zero (10 mmol/L EGTA) to 39 μmol/L (10 mmol/L Ca-EGTA) (Suzuki *et al.* 2014). Fluorescent intensity was recorded with a multi-mode microplate reader (Flexstation 3, Molecular Devices, USA). The excitation/emission wavelength of TurCaMPs was 434/474 nm (10 nm bandpass). The excitation/emission wavelength of GCaMP6m was 485 / 510 nm (10 nm bandpass). The *K*_d_ value and Hill coefficient were calculated by nonlinear fitting with specific binding with Hill slope function using Prism 9.5.

*In vitro* H^+^-titration (Zhao *et al.* 2011). A series of buffer solutions containing 50 μg/mL protein, 30 mmol/L trisodium citrate and 30 mmol/L borax with pH ranging from 4 to 10 were used. To examine the effect of H^+^ binding, each of the aforementioned buffers has two sets, containing either 10 mmol/L CaCl_2_ or 10 mmol/L EGTA. The pH titration was performed with multi-mode microplate reader, the same as those with *K*_d_ measurements.

To obtain the quantum yield (*Φ*), a previously described method was used (Goedhart *et al*. 2012; Li *et al.* 2023). The protein was diluted in buffers containing either 0 or 39 μmol/L CaCl_2_. Firstly, the absorption value of the sample at 430 nm was measured by using an ultraviolet and visible spectrophotometer. Then, the fluorescence emission spectra in the 440–650 nm range were collected, and the spectral area of the sample was calculated by using a FS5 spectrophotometer. Finally, fluorescence intensity and absorbance values were integrated to get the slopes (S) by linear regression of the curve. *Φ* was then calculated as: *Φ*_protein_ = *Φ*_standard_ × (*S*_protein_/*S*_standard_) (Li *et al.* 2023). Reference standard, mTq2 (*Φ* = 0.93) (Goedhart *et al.* 2012).

To obtain the extinction coefficient (*ε*), the alkali-denaturation method was used (Gross *et al.* 2000). Absorbance spectra of purified GECI proteins in buffers containing either 0 or 39 μmol/L CaCl_2_ were measured in triplicate to determine the mean peak absorbance value (*A*). Subsequently, double-concentration samples were denatured by mixing with 2 mol/L NaOH in a 1:1 ratio, and the corresponding mean peak absorbance values (*A*_s_) (with a peak at ~447 nm) were obtained similarly. Assuming the *ε* of the denatured chromophore equivalent to that of a denatured avGFP chromophore (44 (mmol/L)^–1^cm^–1^), the *ε* values of GECIs under apo or Ca^2+^ saturated conditions were then calculated as ε = *A*/*A*_s_*44 (mmol/L)^–1^cm^–1^.

#### Cell culture and gene transfection

Cells were cultured in DMEM (HyClone, Chicago, IL, USA) medium supplemented with 10 % Fetal Bovine Serum (FBS) (AusgeneX, Australia) and 1 % penicillin and streptomycin(P/S) (Thermo Scientific, Waltham, MA, USA) (37 °C, 5 % CO_2_) (Li *et al.* 2020). For gene transfection, plasmids were electroporated into cells with the Bio-Rad Gene Pulser Xcell system (Bio-Rad, Hercules, CA, USA), using voltage step pulse (180 V, 25 ms). After transfection, cells were seeded on round coverslips, first cultured in serum-free OPTI-MEM for 40 min (Thermo Scientific, Waltham, MA, USA), then in regular DMEM medium containing 10 % FBS and 1% P/S for 24 h.

#### Fluorescence imaging

To perform Ca^2+^ imaging, cells were imaged using an inverted ZEISS observer Z1 live-cell imaging system controlled by SlideBook 6.0.23 software (Intelligent Imaging Innovations, Inc.) (Li *et al.* 2023). TurCaMP/mt-TurCaMP, mt-GCaMP6m and mt-pHmScarlet signals were collected with CFP ((438 ± 12 nm)_ex _/ (470 ± 12 nm)_em_), GFP ((470 ± 11 nm)_ex _/(512 ± 13 nm)_em_), and RFP filters ((549 ± 6 nm)_ex _/ (630 ± 50 nm)_em_), respectively. During the Ca^2+^ imaging experiment, cells submerged in imaging solution containing (in mmol/L): NaCl (107), KCl (7.2), MgCl_2_ (1.2), glucose (11.5), and HEPES-NaOH (20), (pH = 7.2). Date was analyzed with MATLAB 2023b (The MathWorks, Natick, MA, USA) and plotted with Prism 9.5. Error bars represent SEM (*n* = 3).

#### Measurements of Basal brightness of GECIs in HEK293 cells

mKate-P2A-GECI plasmids were transfected in HEK293 cells to drive expression of mKate and GECI at nearly equal levels. The fluorescence signals of mKate and TurCaMP / Tq-Ca-FLITs were then collected by the above fluorescence imaging equipment using CFP ((438 ± 12 nm)_ex_ / (470 ± 12 nm)_em_) and RFP filters ((549 ± 6 nm)_ex_ / (630 ± 50 nm)_em_), respectively. Basal fluorescence (*F*_GECI_) was represented as fluorescent ratios (*F*_GECI_ / *F*_mKate_) to eliminate artifacts arising from variations in GECI expression.

#### Assessments of SNRs in HeLa and HEK293 cells

Cells transfected with equal amount of pCDNA3.1-mt-TurCaMPb or -mt-GCaMP6m plasmids were imaged using CFP filters ((438 ± 12 nm)_ex_ / (470 ± 12 nm)_em_) or GFP filters ((470 ± 11 nm)_ex_ / (512 ± 13 nm)_em_), respectively. The excitation light intensity for both GECIs were set the same with the help of a LP10 Laser Power Meter (SANWA, Okayama City, Okayama Prefecture, Japan). After collecting basal fluorescence, ER stores Ca^2+^ stores were first emptied with 2.5 μmol/L iono and 1 μmol/L TG, followed by the addition of 30 mmol/L Ca^2+^ to the extracellular bath to maximize Ca^2+^ concentration within mitochondria via SOCE. The corresponding fluorescence collected under high SOCE conditions were considered as fluorescence under high-Ca^2+^ conditions.

The offset-corrected fluorescence (*F*) readings and the standard deviation (*SD*) values of the image background were measured using Image J software. These values were then converted to electron numbers by multiplying the Gain of the camera used. Therefore, the background noise (*N*) was calculated as N = SD*Gain, and the signal (*S*) in electrons was calculated as S = F*Gain. The SNR was calculated by the following formula:

$ SNR=\frac{S}{\sqrt{{N}^{2}+S}} \;. $

#### Confocal imaging

Confocal imaging was undertaken with a ZEISS LSM880 microscope equipped with a 63x oil objective (NA 1.4) and ZEN 2.1 software. For mt-BFP and mt-TurCaMP, both were excited with a 405 nm laser (0.5% power), and the corresponding fluorescence at 440–510 nm was collected. As to mt-pHmScarlet, fluorescence at 550–670 nm excited with a 543 nm laser (4% power) was collected. The images were analyzed by Image J software.

## Conflict of interest

Wenjia Gu, Yuqin Yang, Yuqing Wang, Jia Li, Wanjie Li, Xiaoyan Zhang, Hao Dong and Youjun Wang declare that they have no conflict of interest.
